# Pelvic Organ Prolapse-Health-Preserving Attitudes According to Sociodemographic Factors

**DOI:** 10.3390/jcm14217863

**Published:** 2025-11-05

**Authors:** Aleksandra Zaborowska, Katarzyna Tomczyk, Małgorzata Kampioni, Paweł Rzymski

**Affiliations:** Department of Mother’s and Child’s Health and Minimally Invasive Gynecology, Gynecologic and Obstetrical University Hospital, Poznan University of Medical Sciences, 61-701 Poznan, Poland

**Keywords:** prolapse, urogynecology, prevention

## Abstract

**Objectives**: Pelvic organ prolapse (POP) disorders are a significant problem with a society-wide dimension, affecting the quality of life of many women around the world. The purpose of this study is to assess the influence of sociodemographic factors on health-preserving behaviors in relation to pelvic organ prolapse in women of reproductive age. **Method**: The survey was conducted using a questionnaire made available electronically and a paper questionnaire distributed to female patients of the Gynecology and Obstetrics Clinical Hospital of the Karol Marcinkowski Medical University in Poznan. In total, 160 women aged 15–49 years voluntarily participated in the study. The distribution of variables was assessed using the Shapiro–Wilk test. The Mann–Whitney U and Kruskal–Wallis ANOVA tests were used for comparisons between groups. Comparisons between assessment scores and self-assessment of knowledge were made using Wilcoxon’s paired rank order test and the Chi2 NW (highest reliability) test. A *p*-value < 0.05 was considered statistically significant. **Results**: The level of knowledge about POP prevention and conservative treatment methods is low. The group with a higher level of knowledge was characterized by younger age, higher education, and living in areas with a large population. **Conclusions**: The results suggest only a partial understanding of the topic of pelvic organ prolapse, while lacking full awareness of prevention.

## 1. Introduction

Pelvic organ prolapse (POP) disorders are a significant problem with a society-wide dimension, affecting the quality of life of many women around the world. Due to their prevalence and physical as well as psychological consequences, they have a significant impact on public health, as well as on economic and social aspects. Despite the huge number of women who struggle with this condition, public awareness remains quite low. The increase in life expectancy and the quest for an improved quality of life are both contributing to a rise in the prevalence of POP, as well as an increase in the number of women seeking treatment and solutions to their symptoms. Currently available demographic data are not precise enough to accurately estimate the true prevalence of POP in the population. However, ongoing efforts by international associations such as the IUGA (International Urogynecological Association) and ICS (International Catheter Solutions) to standardize the diagnosis and treatment of POP may greatly improve our ability to estimate the true incidence and prevalence of this condition in the coming years.

The purpose of this study is to assess the influence of sociodemographic factors on health-preserving behaviors in relation to pelvic organ prolapse in women of reproductive age.

The prevalence of POP is relatively difficult to determine. In the literature, figures range from 3 to 50%. The most recent meta-analysis indicates that 30.9% of the population is affected by POP (95% confidence interval: 24.4–38.2%), with a prevalence of 25.0% observed in subgroups utilizing questionnaire-based methods and 41.8% in subgroups based on gynecological examination as the diagnostic method [1]. POP currently affects between 1 and 65% of women who have given birth in the past. As many as 20% of them require surgical intervention to be performed before the age of 80. Most surgeries are carried out on women aged 60 to 69. Less than 58% of surgeries are performed on women under 60 and the recurrence rate after surgical interventions is as high as 13%. The problem becomes especially apparent during menopause, when a woman’s hormonal status is disrupted, as well as during some gravity sports [2,3,4,5].

The primary theory explaining the etiology of this problem is the so-called integral theory. Its general premise is based on the flaccidity of the ligaments, muscles, and connective tissue of the vagina [4,6,7].

According to its assumptions, there are three main levels of organ decline:

- Anterior vaginal wall/cystocele—this is the most common form of prolapse of the reproductive organs. It refers to the lowering of the urinary bladder. This defect is associated with the highest rate of recurrence, despite the surgical treatment applied.
- Posterior wall—mainly refers to rectocele (hernia of the rectum).
- Top defect—involves uterine prolapse and lowering of the cervix as well as the vaginal stump after a previous hysterectomy. In addition, it can involve the small intestine, colon, and bladder [4].

The complexity of this problem is also due to the vast number of possible causes and risk factors. We distinguish between these causes:

- Functional—disorders that prevent normal functioning;
- Anatomical—abnormal anatomical structure;
- Neurogenic—a disorder in the normal functioning of the nervous system, which directs the work of internal organs;
- Iatrogenic [8].

A number of factors affecting the incidence of POP have been described in the literature. However, a key role is attributed to weakness of the muscles and connective tissue surrounding the pelvic organs. The correct interaction between these two factors ensures optimal support of the internal organs. When the pelvic floor muscles are dysfunctional, the strength of the vaginal walls is weakened in response to changing pressure, thus causing abnormal tension on the ligamentous attachments to the lateral pelvic walls [7,9].

There are many factors that can be responsible for the weakening of the muscular apparatus. Among them, intense physical exertion and overexertion are often mentioned. Another possible mechanism includes the abdominal muscle complex dyssynergy with PFM (Pelvic Floor Muscles) [5,7]. Women who have given birth by natural childbirth are at a higher risk of such complications. Compared to non-births, the risk increases two to eight times. The risk of POP is four times higher after the first natural childbirth, eight times after the second and nine times after the third. Childbirth requires an intense effort from the muscles, which are constantly working during labor, contracting and then stretching again. The fetus’s head, as it descends in the birth canal, causes tremendous pressure and friction on the surrounding tissues. The risk of damage is nine times higher with a natural childbirth, compared to a cesarean section. The most common damage associated with natural childbirth is observed in the pubococcygeal part of the anal lever muscle. Visualization of the course of labor shows that during the second period of labor, the anal lever muscle can be stretched by as much as 200% compared to its original length. In 55% of women with prolapse of the reproductive organ, damage is found in this very muscle. This is associated with an excessive increase in pressure and strong pushing of the parturient [9,10].

Other pre- and perinatal factors that increase the risk of POP are as follows:

- Baby’s birth weight > 4000 g.
- Mother’s age at birth > 35.
- Maternal BMI > 25 kg/m^2^.
- Operative deliveries: using forceps, or vacuum aspiration.
- Prolonged labor.
- Episiotomy, especially central and mid-lateral incision < 60° [10].

Studies show the positive impact of introducing physiotherapy in a structured manner early in pregnancy. Such practices can lead to a dramatic decrease in the incidence of incontinence by as much as 62% during pregnancy and by 29% in the 3–6-month postpartum period. However, in spite of the research, there is still insufficient data on the effectiveness of such exercises in the postpartum period.

With women’s life expectancy steadily increasing, the incidence of POP is expected to increase in direct proportion in the coming years. It is believed that by 2050, the number of women with POP in the US will have increased by about 46% [6,9,11].

As we know, genetic predisposition plays an important role in susceptibility to various diseases, affecting the risk of developing them. It has been proven that genetic predisposition can increase the risk of POP by up to 2.5-times compared to the general population. Reduced collagen content in tissues is associated not only with menopause, but also with obesity, or vascular diseases. Particular attention is paid to the occurrence of varicose veins, embolism, rheumatic diseases, not only in a particular patient, but also in her first- and second-degree relatives. Genetic predisposition is also responsible for the type and “quality” of collagen produced [6,12].

## 2. Material and Methods

This survey was conducted using a questionnaire made available electronically and a paper questionnaire distributed to female patients of the Heliodor Święcicki Gynecology and Obstetrics Clinical Hospital of the Karol Marcinkowski Medical University in Poznan. Patients completed the questionnaire during their hospitalization or within 14 days of being discharged, via the internet. Patients in both groups (those in hospital and online) completed the same questionnaires to minimize the potential risk of selection bias. Participation in the study was voluntary; the respondents were informed of the anonymity of the survey. Written informed consent was obtained from all participants, and the study protocol was approved by the CEO of Heliodor Święcicki Gynecology and Obstetrics Clinical Hospital of the Karol Marcinkowski Medical University in Poznan (11/F01/2024/KZMD, 1 November 2024). In total, 160 women participated in the study. The inclusion criterium of the study was the age of the women, from 15 to 49 years old. The patients were recruited between 1 November 2024 and 31 December 2024.

The questionnaire used in this study was non-validated and consisted of 30 questions which included questions about age, residence, education, weight, height, presence of concomitant diseases, use of hormone therapy, and obstetric history. In a further section, questions focused on the presence or absence of complaints of pelvic organ prolapse, urinary incontinence, and on knowledge of methods of prevention and treatment of genital static disorders.

To analyze the collected data, Statistica (Cloud Software Group, Inc. (2023 Fort Lauderdale, FL, USA), Data Science Workbench, version 14) and Microsoft Excel (Microsoft Office (2019), version 2205) were used. The data did not follow the normal distribution; thus, non-parametric tests were applied. The distribution of variables was assessed using the Shapiro–Wilk test. The Mann–Whitney U and Kruskal–Wallis ANOVA tests were used for comparisons between groups. Comparisons between assessment scores and self-assessment of knowledge were made using Wilcoxon’s paired rank order test. Using the Chi^2^ NW (highest reliability) test, an examination of the relationship between variables was conducted. The confidence level used in all calculations was *p* < 0.05.

## 3. Results

The characteristics of the study group are shown in Table 1 and Table 2.

Significantly fewer visits to a gynecologist were recorded in women who struggled with reproductive organ prolapse (*p* < 0.001) (Table 3).

The analysis shows that the use of pelvic floor physical therapy is more frequent among patients with higher education and women living in larger cities (>50,000 residents). In addition, a correlation between POP and greater interest in pelvic floor physical therapy was observed (Table 4).

Table 5 illustrates the statistical analysis concerning the performance of exercises to strengthen the muscles of the lower pelvic floor, depending on the following variables: age, education, place of residence, having undergone natural childbirth, POP, and urine incontinence.

As many as 60% of female respondents declare having some knowledge of the characteristics of the work of a pelvic floor physical therapist. The remainder of the group—40%—had no knowledge of this subject (Table 6).

Significant differences were observed in the answers posed depending on the age of the patients, education, and place of residence. Younger respondents, i.e., <35 years of age, declared greater knowledge of the characteristics of the work of a pelvic floor physical therapist (71.23%) significantly more often than women >35 years of age, among whom the percentage was 50.57%. Marked discrepancies are also observed between respondents with a university education (in this group, as many as 84.09% of respondents declared knowledge of the subject matter) and women with education other than university (the percentage was 30.65%). A similar relationship applies to place of residence. For those living in localities with a population of >50,000, 78.75% confirmed their familiarity with the subject under study, while only 41.25% of women living in smaller localities did so.

In Table 7, greater awareness of the topic of preventive methods for reproductive organ prolapse is observed among women with higher education and among women living in towns with a population of >50,000. For both relationships, the confidence level was less than 0.001.

The vast majority of patients, 77.50% (n = 124), deny knowledge of the therapeutic method, which is pessarotherapy. Only 22.50% (n = 36) have some awareness of it, as seen in Table 8.

People who significantly more often reported having had a recent visit to a gynecologist within the last year were women under 35 years of age, with higher education, living in towns with a population exceeding 50,000, women who had not experienced natural childbirth, and women not struggling with the problem of genital prolapse/urinary incontinence.

Significantly less frequent visits to the gynecologist were observed in women struggling with genital prolapse (*p* < 0.001) (Table 3).

Pelvic floor muscle exercises are performed more frequently by women with higher education and those who live in cities with a population of over 50,000 (Table 4).

A higher frequency of pelvic floor muscle exercises is observed in women with higher education, living in cities with a population of over 50,000, and in respondents suffering from POP (Table 5).

Younger respondents, under 35, demonstrate greater knowledge of the characteristics of the work of a pelvic floor physical therapist (71.23%) compared to women over 35, among whom the percentage was 50.57%. Marked discrepancies were also observed between respondents with higher education (in this group, as many as 84.09% of respondents declared knowledge of the subject) and women with education other than higher education (this percentage was 30.65%). A similar relationship applies to place of residence. People living in towns with a population of over 50,000 confirmed their knowledge of the subject in 78.75% of cases, while women living in smaller towns did so in only 41.25% of cases.

Women with higher education and women living in towns with a population of over 50,000 have greater knowledge of preventive methods for POP. For both correlations, the confidence level was less than 0.001.

A low level of knowledge about pessarotherapy was observed among respondents. The results suggest that higher education and place of residence (>50,000 inhabitants) have a positive impact on the level of knowledge in this area. In addition, women struggling with urinary incontinence are more aware of this method (Table 8).

## 4. Conclusions

1. The level of knowledge about POP prevention and conservative treatment methods is low.
2. Differences in the level of knowledge held depend primarily on factors such as age, place of residence, and type of education.
3. The non-validated questionnaire that was used in the study may result in unreliable data collection and make it difficult to compare data with that of other authors. The data collection method (using both paper and digital version of the survey) in addition to the single-center nature of the study serve as limitations of the study. We may assume that the results are preliminary rather than definitive.

## 5. Discussion

Pelvic organ prolapse POP is affecting an increasing number of women around the world. Epidemiological forecasts in recent medical studies clearly predict a rise in the number of women experiencing this problem in the future. This is largely due to the increasing average age of the population, the lifestyle we lead, as well as other environmental factors [6]. In view of this, it seems crucial to spread knowledge regarding both the prevention and treatment of female pelvic organ prolapse.

The results of published studies clearly indicate a low level of knowledge in the general female population regarding pelvic floor muscle dysfunction, risk factors, as well as treatment options [6,8].

The results of the present study suggest only a partial understanding of the topic of pelvic organ prolapse, while lacking full awareness of prevention methods. Similar correlations can be observed in other scientific publications. As many as 81% of women participating in a survey conducted by the Brazilian Society of Gynecologists and Obstetricians declared that they had never received any information about the possibility of this type of disorder. As in our study, the group with a higher level of knowledge was characterized by higher education and living in areas with a large population [13].

Demographic variables prove to have a significant impact on the state of awareness and knowledge of the women surveyed. The difference in the level of knowledge between respondents with higher education and women with lower education was almost 32%. A similar observation was noted in a pilot study conducted by the International Urogynecology Journal in 2022. Younger women and those with higher education had a higher percentage of correct answers in the questionnaire on general knowledge about female reproductive organs and prolapse [14]. The same trend is observed in knowledge regarding risk factors. Those living in larger cities show greater awareness of medical factors such as menopause, congenital spinal defects, and obesity. The most easily recognized factor was natural childbirth. Knowledge of risk factors greatly influences patients’ lifestyles and the use of health-preserving behaviors. A noticeable problem is the presence of myths and misconceptions among patients. African-American ethnicity, limited access to information, low levels of education, and low socioeconomic status are cited as major risk factors that correlate with the level of low knowledge described in other articles. In contrast to this analysis, a study conducted by the Brazilian Society of Gynecologists and Obstetricians observed greater knowledge of POP pathophysiology among women suffering from the disorder [13]. The data we obtained show a higher frequency of this group of women identifying inappropriate behaviors as preventive methods. It seems education may act as an indirect factor for POP through less knowledge and awareness of prevention (e.g., pelvic floor muscle exercises), greater parity, poorer access to healthcare, physical work that strains the pelvic floor, and lifestyle factors (obesity, smoking).

The subject of female reproductive organs prolapse is still a taboo. Conducting conversations on this topic requires respecting women’s privacy, maintaining discretion, and building trust between the person imparting knowledge and the patient. Despite public respect towards doctors, patients are relatively often not satisfied with their services. It has been shown in the literature that women often experience belittling of the problem, ignorance, and a dismissive approach to the complaints they report [15]. A small percentage of women consult gynecological problems with health professionals. On average, only 50% of the female population who suffer from genital static disorders consult a healthcare professional. As identified, the reasons for such behavior are shame, lack of knowledge, and belittling the problem [13]. Many of them also suffer from sexual disorders [16,17]. A dismissive attitude towards the patient results in them giving up trying to seek help. This, in turn, leads to an exacerbation of symptoms and, ultimately, a reduction in the woman’s quality of life.

The subject of preventive measures in this area is developing rapidly. There are many methods that can be highly effective. Recent scientific studies have pointed to the effectiveness of measures which do not require much effort, i.e., changing lifestyle, diet, quitting smoking, avoiding lifting heavy loads or performing physically demanding work [18]. The most popular preventive methods, and at the same time the methods with demonstrated effectiveness in alleviating changes resulting from genital static disorders, are pelvic floor muscle exercises [5,7,19]. Despite the fact that more than half of the surveyed group agrees with the statement that preventive methods can have a positive effect on symptoms associated with pelvic organ prolapse, the percentage of patients using these methods is low. Improving women’s awareness of implementable preventive measures, as well as familiarizing them with pelvic floor muscle exercises, could reduce the number of cases of POP or alleviate the severity of current lesions. The data collected during the survey unequivocally shows that more than half of the surveyed group has little or no knowledge of POP prevention methods. In total, 60% of female respondents declare a knowledge of the characteristics of the work of a pelvic floor physical therapist. At the same time, a similar percentage of respondents—63%—have never performed exercises to strengthen their pelvic floor muscles [5,7]. A higher frequency of performed exercises is observed only in women who are younger, have higher education, and live in larger cities. The same relationship is observed in respondents who gave birth by a natural method. However, no significant differences are noted in the frequency of exercise in relation to the age of the patient, urine incontinence, or pelvic organ prolapse. Similar findings are observed in medical articles, which report that women with higher education are more likely to exercise as well as women with a history of consultation with medical personnel. Correlations of low significance, however, were observed between knowledge of the pelvic floor muscles and the age of the patient, as well as experience of natural childbirth [13,14]. The correlations presented in the given articles coincide with the conclusions gathered in the study. The question that arises at this point is as follows: what changes need to be made to achieve improvements in this area? We need to extend assistance to women in each variability group. It would seem beneficial to introduce free educational classes on pelvic floor muscle exercises. Such classes could be held at hospitals, or clinics easily accessible to patients from both smaller and larger towns. Alarming data is noted on the issue of pessarotherapy, which is currently growing in popularity, mainly due to its non-invasiveness, ease of use and the positive benefits of its use [20]. As many as 77% of the women surveyed are not familiar with this method, which could reduce the discomfort they experience on a daily basis. Again, greater familiarity with preventive methods is observed among women who are better educated, live in larger cities, but also those who struggle with urine incontinence. Female respondents also show moderate knowledge of appropriate forms of physical activity in the context of preventive measures. Although pelvic floor muscle exercises, yoga, and Pilates are recognized as beneficial, some women still choose intense forms of activity that can negatively affect the pelvic floor. It is recommended to promote moderate forms of exercise, such as leisurely cycling and swimming, which promote health by leveling excessive strain on pelvic structures.

The problem of pelvic organ prolapse significantly affects the daily activity of women. These changes limit their social life, participation in sports and even family life. Less frequent visits to the gynecologist are observed in this group of women, which may be due to a sense of shame or embarrassment, among other reasons. POP has a significant impact on women’s sex life. The resulting anatomical changes may be associated with discomfort during intercourse, and even with pain, which can result in a woman’s isolation, a drastic drop in self-esteem, a decrease in sexual desire, as well as a weakening of the relationship with her partner due to concerns about pleasing her partner and a sense of shame. The sexual needs of female patients should not be forgotten. In the general female population, about 30–40% report sexual dysfunction due to POP. However, in women suffering from pelvic floor disorders, the percentage rises to as high as 50–83% [21]. Patients struggling with pelvic organ prolapse also experience changes related to decreased lubrication of the vaginal tissues and loosening of the vaginal walls. It is also worth mentioning the less frequent achievement of orgasm in these patients. It is believed that the negative impact of POP on sexual life does not depend mainly on the resulting anatomical changes, but on psychological factors. In this group of women, there is a higher risk of depression compared to the general population. Depression affects between 20 and 71% of women in this case [22]. Giving up intimacy is mainly associated with a disturbed perception of one’s own body.

The results directly indicate the need for increased educational efforts. It is very important to share knowledge of preventive methods among all women, especially young women, because they should take care of their pelvic floor muscles during pregnancy and after childbirth. Thus, gynecologists should certainly be more educational during routine checks, paying particular attention to this group of women. Education for menopausal patients could be provided during rehabilitation at health resorts. In Poland, urogynaecological education is now systematically introduced in childbirth classes, internet campaigns, and through education grants from the Polish Physiotherapy Society. A lack of basic knowledge of the pathophysiology of lesions that arise in the reproductive organs, as well as their potential health consequences and complications, is a significant issue. This may be responsible for the delay in the diagnosis of POP, or the introduction of inappropriate treatment.

## Figures and Tables

**Table 1:** Characteristics of the study group.

Respondent Characteristics		Percentage [%]
Level of education	Higher education	55
	Other	45
Location residence	<50,000 citizens	50
	>50,000 citizens	50
Natural childbirth	No	39
	One	23
	More than one	38
Pelvic organ prolapse	Yes	32
	No	68
pelvic floor physical therapist frequency	Never	79
	Once	8
	Several times	13
Frequency of pelvic floor muscle exercises	No	60
	At least 2 times a week	11
	At least several times a month	29

**Table 2:** Anthropometric measurements in the study group of women.

	N	M ± SD	Min–Max	Me [Q1–Q3]
Age	160	36.4 ± 9.85	16–49	36 [29–46]
Growth	160	166.5 ± 5.98	149–182	166 [163–170.5]
Body weight	160	71.12 ± 14.31	45–115	69.5 [60–81]
BMI	160	25.68 ± 5.22	16.94–40.51	24.9 [21.8–29.07]

**Table 3:** Influence of demographic factors on the timing of the last visit to a gynecologist.

		Last Visit to the Gynecologist				*χ* ^2^	*p*
		<1 Year (50%)	1–5 Years (33.13%)	>5 Years (9.38%)	I Don’t Remember (7.5%)		
Age	<35 years	49 (67.12%)	24 (32.88%)	0 (0%)	0 (0%)	**40.76**	**<0.001**
	>35 years	31 (35.63%)	29 (33.33%)	15 (17.24%)	12 (13.79%)		
Education	Higher	51 (57.95%)	34 (38.64%)	2 (2.27%)	1 (1.14%)	**27.6**	**<0.001**
	Other	29 (40.28%)	19 (26.39%)	13 (18.06%)	11 (15.28%)		
Location residence	<50 k.	34 (42.5%)	20 (25%)	14 (17.5%)	12 (15%)	**35.11**	**<0.001**
	>50,000	46 (57.5%)	33 (41.25%)	1 (1.25%)	0 (0%)		
Childbirth natural	At all	42 (66.67%)	20 (31.75%)	0 (0%)	1 (1.59%)	**33.3**	**<0.001**
	One	17 (47.22%)	14 (38.89%)	4 (11.11%)	1 (2.78%)		
	More	21 (34.43%)	19 (31.15%)	11 (18.03%)	10 (16.39%)		
pelvic organ prolapse	Yes	14 (27.45%)	17 (33.33%)	9 (17.65%)	11 (21.57%)	**32.52**	**<0.001**
	Not	66 (60.55%)	36 (33.03%)	6 (5.5%)	1 (0.92%)		
incontinence urinary	Yes	27 (36.99%)	28 (38.36%)	11 (15.07%)	7 (9.59%)	**11.28**	**0.01**
	Not	53 (60.92%)	25 (28.74%)	4 (4.6%)	5 (5.75%)		

bold values are statistical significant *p* < 0.05.

**Table 4:** Frequency of use of pelvic floor physical therapist according to demographic factors.

		Use of a Pelvic Floor Physical Therapist			*χ* ^2^	*p*
		Never	Once	Several Times		
Age	<35 years	56 (76.71%)	6 (8.22%)	11 (15.07%)	0.83	0.66
	>35 years	71 (81.61%)	7 (8.05%)	9 (10.34%)		
Education	Higher	59 (67.05%)	10 (11.36%)	19 (21.59%)	**22.8**	**<0.001**
	Other	68 (94.44%)	3 (4.17%)	1 (1.39%)		
Location residence	<50 k.	71 (88.75%)	3 (3.75%)	6 (7.5%)	**9.04**	**0.01**
	>50,000	56 (70%)	10 (12.5%)	14 (17.5%)		
Childbirth natural	No	54 (85.71%)	6 (9.52%)	3 (4.76%)	9.11	0.06
	One	28 (77.78%)	4 (11.11%)	4 (11.11%)		
	More	45 (73.77%)	3 (4.92%)	13 (21.31%)		
Pelvic organ prolapse	Yes	36 (70.59%)	3 (5.88%)	12 (23.53%)	**7.9**	**0.02**
	No	91 (83.49%)	10 (9.17%)	8 (7.34%)		
Incontinence urinary	Yes	54 (73.97%)	5 (6.85%)	14 (19.18%)	5.62	0.06
	No	73 (83.91%)	8 (9.2%)	6 (6.9%)		

bold values are statistical significant *p* < 0.05.

**Table 5:** Performance of pelvic floor muscle exercises according to demographic factors: A. not at all; B. several times a year; C. several times a month; D. two–three times a week; E. every day.

		Performing Exercises for the Muscles of the Lower Pelvic Floor					*χ* ^2^	*p*
		A	B	C	D	E		
Age	<35 years	47 (64.38%)	10 (13.7%)	8 (10.96%)	6 (8.22%)	2 (2.74%)	1.8	0.77
	>35 years	50 (57.47%)	17 (19.54%)	11 (12.64%)	8 (9.2%)	1 (1.15%)		
Education	Higher	41 (46.59%)	18 (20.45%)	15 (17.05%)	11 (12.5%)	3 (3.41%)	**19.59**	**0.001**
	Other	56 (77.78%)	9 (12.5%)	4 (5.56%)	3 (4.17%)	0 (0%)		
Location residence	<50 k.	61 (76.25%)	11 (13.75%)	1 (1.25%)	6 (7.5%)	1 (1.25%)	**26.58**	**<0.001**
	>50,000	36 (45%)	16 (20%)	18 (22.5%)	8 (10%)	2 (2.5%)		
Childbirth natural	At all	40 (63.49%)	14 (22.22%)	4 (6.35%)	4 (6.35%)	1 (1.59%)	**15.56**	**0.049**
	One	22 (61.11%)	2 (5.56%)	8 (22.22%)	2 (5.56%)	2 (5.56%)		
	More	35 (57.38%)	11 (18.03%)	7 (11.48%)	8 (13.11%)	0 (0%)		
Pelvic organ prolapse	Yes	31 (60.78%)	7 (13.73%)	5 (9.8%)	7 (13.73%)	1 (1.96%)	2.71	0.61
	No	66 (60.55%)	20 (18.35%)	14 (12.84%)	7 (6.42%)	2 (1.83%)		
Incontinence urinary	Yes	41 (56.16%)	13 (17.81%)	9 (12.33%)	9 (12.33%)	1 (1.37%)	2.69	0.61
	No	56 (64.37%)	14 (16.09%)	10 (11.49%)	5 (5.75%)	2 (2.3%)		

bold values are statistical significant *p* < 0.05.

**Table 6:** Knowledge of pelvic floor physical therapy according to demographic characteristics of respondents.

		Pelvic Floor Physical Therapy		*χ* ^2^	*p*
		Yes	No		
Age	<35 years	52 (71.23%)	21 (28.77%)	**7.16**	**0.01**
	>35 years	44 (50.57%)	43 (49.43%)		
Education	Higher	74 (84.09%)	14 (15.91%)	**49.62**	**<0.001**
	Other	22 (30.56%)	50 (69.44%)		
Location residence	<50 k.	33 (41.25%)	47 (58.75%)	**24.16**	**<0.001**
	>50,000	63 (78.75%)	17 (21.25%)		
Childbirth natural	At all	42 (66.67%)	21 (33.33%)	2.63	0.27
	One	22 (61.11%)	14 (38.89%)		
	More	32 (52.46%)	29 (47.54%)		
Pelvic organ prolapse	Yes	25 (49.02%)	26 (50.98%)	3.73	0.054
	Not	71 (65.14%)	38 (34.86%)		
Incontinence urinary	Yes	39 (53.42%)	34 (46.58%)	2.42	0.12
	Not	57 (65.52%)	30 (34.48%)		

bold values are statistical significant *p* < 0.05.

**Table 7:** Knowledge of preventive methods of POP according to demographic characteristics of respondents.

		Prevention of POP		*χ^2^*	*p*
		Yes	Not		
Age	<35 years	30 (41.1%)	43 (58.9%)	0.23	0.63
	>35 years	39 (44.83%)	48 (55.17%)		
Education	Higher	56 (63.64%)	32 (36.36%)	**35.41**	**<0.001**
	Other	13 (18.06%)	59 (81.94%)		
Location residence	<50 k.	18 (22.5%)	62 (77.5%)	**28.69**	**<0.001**
	>50,000	51 (63.75%)	29 (36.25%)		
Childbirth natural	No	25 (39.68%)	38 (60.32%)	0.82	0.66
	One	15 (41.67%)	21 (58.33%)		
	More than one	29 (47.54%)	32 (52.46%)		
Pelvic organ prolapse	Yes	23 (45.1%)	28 (54.9%)	0.12	0.73
	Not	46 (42.2%)	63 (57.8%)		
Incontinence urinary	Yes	29 (39.73%)	44 (60.27%)	0.63	0.43
	Not	40 (45.98%)	47 (54.02%)		

bold values are statistical significant *p* < 0.05.

**Table 8:** Influence of demographic factors on knowledge of pessarotherapy among respondents.

		Pessarotherapy		*χ* ^2^	*p*
		Yes	Not		
Age	<35 years	19 (26.03%)	54 (73.97%)	0.95	0.33
	>35 years	17 (19.54%)	70 (80.46%)		
Education	Higher	31 (35.23%)	57 (64.77%)	**20.1**	**<0.001**
	Other	5 (6.94%)	67 (93.06%)		
Location residence	<50 k.	8 (10%)	72 (90%)	**15.01**	**<0.001**
	>50,000	28 (35%)	52 (65%)		
Childbirth natural	At all	18 (28.57%)	45 (71.43%)	2.19	0.33
	One	7 (19.44%)	29 (80.56%)		
	More	11 (18.03%)	50 (81.97%)		
Pelvic organ prolapse	Yes	8 (15.69%)	43 (84.31%)	2.09	0.15
	No	28 (25.69%)	81 (74.31%)		
Incontinence urinary	Yes	10 (13.7%)	63 (86.3%)	**6.17**	**0.01**
	No	26 (29.89%)	61 (70.11%)		

bold values are statistical significant *p* < 0.05.

## Data Availability

The data presented in this study are available on request from the corresponding author due to privacy.
